# Supplementary material for: Treponema denticola Induces Neuronal Apoptosis by Promoting Amyloid-β Accumulation in Mice
Source: Pathogens. 2022 Oct 5;11(10):1150. doi: 10.3390/pathogens11101150 (PMC9610539; doi:10.3390/pathogens11101150)
Supplement: Supplementary file 1 [file pathogens-11-01150-s001.zip › pathogens-1939976-supplementary.pdf]

Supplementary Materials

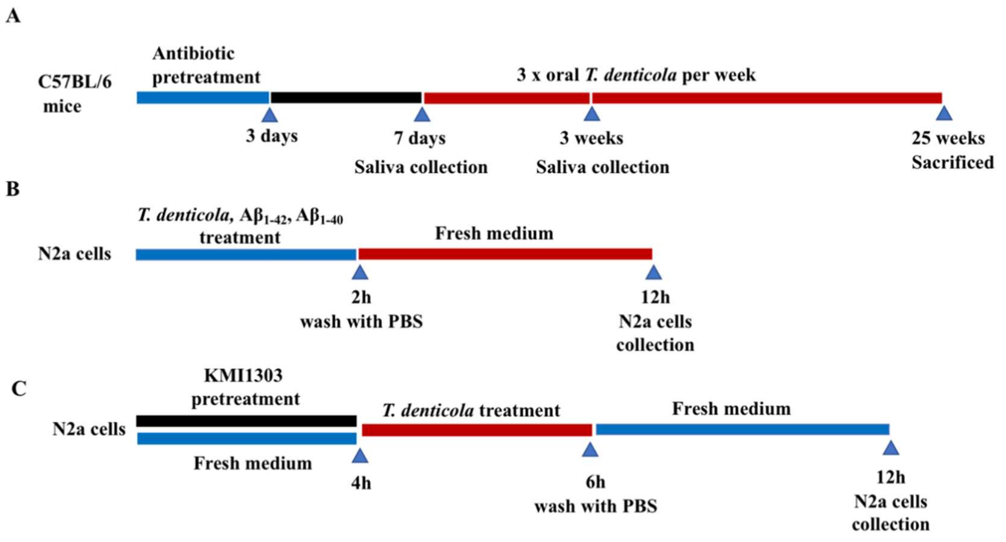

Figure S1. Study design. (A) Protocol used to construct *T. denticola* and *P. gingivalis* oral infection models. (B) Protocol used to quantitatively analyze the effect of the *T. denticola,* $A\beta_{1-40}$, and $A\beta_{1-42}$-induced apoptosis in N2a cells. (C) Protocol used to quantitatively analyze the effect of Aβ inhibition on apoptosis caused by *T. denticola*-infection in N2a cells.

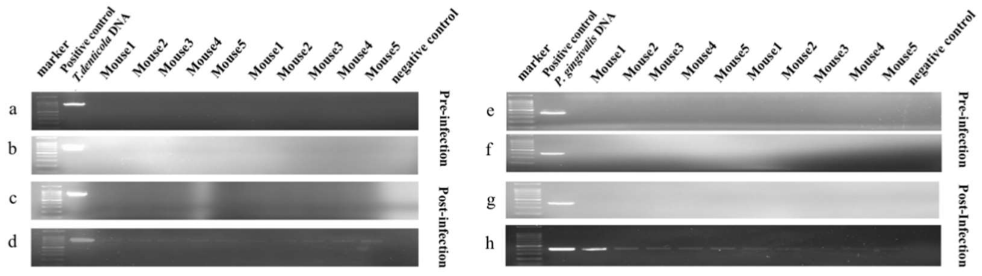

Figure S2. Bacteria detection (determined by PCR) in the saliva of mice. (Pre-infection: a, e: sham group; b: *T. denticola* group; f: *P. gingivalis* group; Post-infection: c, g: sham group; d: *T. denticola* group; h: *P. gingivalis* group).
